# Anti-Biofilm Performance of Three Natural Products against Initial Bacterial Attachment

**DOI:** 10.3390/ijms141121757

**Published:** 2013-11-04

**Authors:** Maria Salta, Julian A. Wharton, Simon P. Dennington, Paul Stoodley, Keith R. Stokes

**Affiliations:** 1 National Centre for Advanced Tribology at Southampton (nCATS), Engineering Sciences, University of Southampton, Highfield, Southampton SO17 1BJ, UK; E-Mails: j.a.wharton@soton.ac.uk (J.A.W.); s.p.dennington@soton.ac.uk (S.P.D.); p.stoodley@soton.ac.uk (P.S.); krstokes@dstl.gov.uk (K.R.S.); 2 Physical Sciences Department, the Defence Science and Technology Laboratory, Porton Down, Salisbury, Wiltshire SP4 0JQ, UK

**Keywords:** marine bacteria, natural products, anti-biofilm, bacterial attachment

## Abstract

Marine bacteria contribute significantly towards the fouling consortium, both directly (modern foul release coatings fail to prevent “slime” attachment) and indirectly (biofilms often excrete chemical cues that attract macrofouling settlement). This study assessed the natural product anti-biofilm performance of an extract of the seaweed, *Chondrus crispus*, and two isolated compounds from terrestrial sources, (+)-usnic acid and juglone, against two marine biofilm forming bacteria, *Cobetia marina* and *Marinobacter hydrocarbonoclasticus*. Bioassays were developed using quantitative imaging and fluorescent labelling to test the natural products over a range of concentrations against initial bacterial attachment. All natural products affected bacterial attachment; however, juglone demonstrated the best anti-biofilm performance against both bacterial species at a concentration range between 5–20 ppm. In addition, for the first time, a dose-dependent inhibition (hormetic) response was observed for natural products against marine biofilm forming bacteria.

## 1. Introduction

The underwater hull of a ship is exposed not only to the corrosive seawater environment, but also to the constant accumulation of biofouling. Biofouling, or marine growth, includes any attaching organisms, such as biofilms (mainly bacteria and diatoms), tubeworms, mussels, barnacles and algae.

Overall, biofouling is a major concern for submersed manmade structures, while, especially, vessel performance can be severely affected in terms of speed, hydrodynamic efficiency, fuel consumption and weight. Marine biofilms, which are mainly comprised of bacteria and diatoms embedded in an extracellular matrix [1], constitute a major component of the overall biofouling and may lead to a 14% increase in ship fuel costs, while an 8%–29% penalty in propulsive power has been attributed to a mature marine biofilm [2,3].

Previously, biofouling has been controlled using toxic coatings, which have subsequently been shown to indiscriminately affect marine life. For instance, tributyltin (TBT) was widely used in coatings for its antifouling (AF) capacity. However, in September, 2008, further applications of TBT coatings were prohibited, in a treaty ratified by the International Maritime Organisation (IMO), due to its toxic effects in the wider marine environment, causing shell deformations in oysters, sex changes (imposex) in whelks and immune response, neurotoxic and genetic effects in other marine species [4]. TBT has been described as the most toxic substance ever deliberately introduced into the marine environment [4,5]. Therefore, the need for new, effective and environmentally friendly coatings has been the focus and challenge for the scientific community. The prohibition of the use of these toxic antifoulants has led to the search for bio-inspired AF strategies. Current attempts towards the production of alternative environmentally benign coatings involve biomimetic approaches [6,7], such as mimicking surfaces of marine organisms that are inherently foul-free and/or incorporating natural AF compounds into marine paints. It is estimated that AF coatings provide the shipping industry with annual fuel savings of $60 billion and reduced emissions of 384 million and 3.6 million tonnes, respectively, for carbon dioxide and sulphur dioxide per annum [8].

In the marine environment, a wide variety of species demonstrate antifouling abilities by several means, e.g., the use of chemical [9] and physical [10] defences or symbiotic relationships between hosts (e.g., algae) and epibionts (e.g., bacteria) that prevent fouling. The inhibition of biofouling in a natural way, as observed in marine organisms, has triggered scientific interest, leading to the examination of marine natural products (NPs) as a possible basis for novel antifouling technology. Since the early 1980s, a great number of marine NPs have been assayed against organisms implicated in the biofouling process, and several reviews dealing with their potential use as novel antifouling biocides have been published [11–15]. Specifically, red algae (phylum: Rhodophyta) are known to exhibit antifouling properties [16,17]. It has also been reported that the red alga, *C. crispus*, exhibits antifouling activity against biofoulers [18,19]. While most studies have concentrated on marine eukaryotes as sources of NPs, bacteria have also been used by incorporating directly into coatings [20] and by extracting enzymes from non-marine isolates [21]. Recently, terrestrial NPs, pharmaceuticals and enzymes have been recognized as important sources of non-toxic antifoulants extensively reviewed by Qian *et al.* [22].

In the current work, three NPs were investigated for their anti-biofilm performance against two model biofilm forming bacteria (*Cobetia marina* and *Marinobacter hydrocarbonoclasticus*). A crude extract of the red seaweed, *Chondrus crispus*, was the marine NP tested, as it had been shown to have anti-microbial efficacy [23], while the terrestrial NPs were the pure compounds, (+)-usnic acid and juglone, isolated from a lichen and the black walnut, respectively n, which have been shown to have antibacterial efficacy [24,25]. The terrestrial products chosen for their AF potential have previously demonstrated antibacterial properties against medically relevant bacteria; however, their activity against marine biofilm forming species is reported here for the first time. The potential for antimicrobial attachment and biofilm formation was quantified through the *in situ* assessment of initial colonization of bacteria using imaging microplate reader technology combined with nucleic acid staining and epifluorescence microscopy.

## 2. Results and Discussion

### 2.1. Growth Conditions (ASW *vs.* NSW)

**Figure 1:**
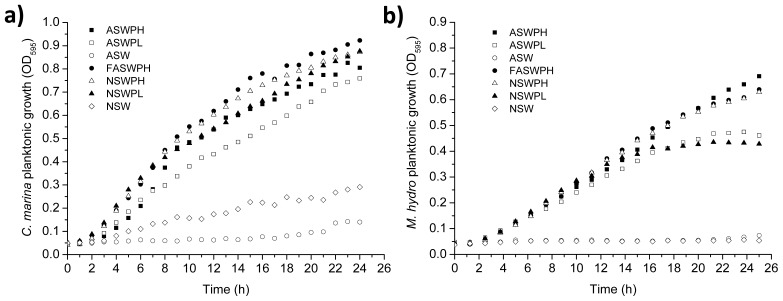
Bacterial growth kinetics over 24 h under different media, using optical density at λ = 595 nm (OD_595_), where: (**a**) *Cobetia marina* and (**b**) *Marinobacter hydrocarbonoclasticus*. ASWPH, artificial sea water peptone high; ASWPL, artificial sea water peptone low; ASW, artificial sea water; FASWPH, filtered artificial sea water peptone high; NSWPH, natural sea water peptone high; NSWPL, natural sea water peptone low; and NSW, natural sea water.

The initial attachment and growth conditions play a key role in cell biology, since the environment may affect the physiology and, therefore, the growth and kinetic response of an organism. Growth rates and doubling times for *C. marina* and *M. hydrocarbonoclasticus* grown under different media are shown in Table 1, while the growth kinetics can be seen in Figure 1. Firstly, we assessed whether it was reasonable to use artificial sea water (ASW) rather than natural sea water (NSW) for subsequent experiments. ASW has the advantage in that it is reproducible and not affected by seasonal or local manmade or natural environmental variations. Overall, when comparing equivalent peptone concentrations for the two different sea waters (ASW *vs*. NSW), no significant differences in planktonic growth rate were found. Therefore we concluded that using ASW as an alternative to NSW was acceptable to avoid seasonal and day-to-day variability in chemistry and nutrients. Regarding the peptone levels for the two media, it was found that the high peptone concentrations (artificial sea water peptone high (ASWPH), 18 g L^−1^) promoted higher bacterial growth when compared to the low concentrations (artificial sea water peptone low (ASWPL), 9 g L^−1^). Specifically, for *C. marina*, the growth rate was significantly higher for ASWPH when compared to ASWPL (*p* < 0.022) and ASW (*p* < 0.001). Similarly, for *M. hydrocarbonoclasticus*, the media with high peptone concentrations (ASWPH) illustrated the best growth performance (ASWPL, *p* < 0.001, and ASW, *p* < 0.001), *i.e.*, contributing to the fastest growth rate. Furthermore, when comparing the sterilization procedures conducted on the ASW media, *i.e.*, filtered *vs*. autoclaved, no significant difference in growth for both species was found. In the literature, autoclaving is routinely used as a medium sterilisation method for bacterial growth; however, to avoid changes in media chemistry, such as pH following autoclaving, the use of the filtration was decided to be the most appropriate procedure for the purposes of this work.

**Figure 2:**
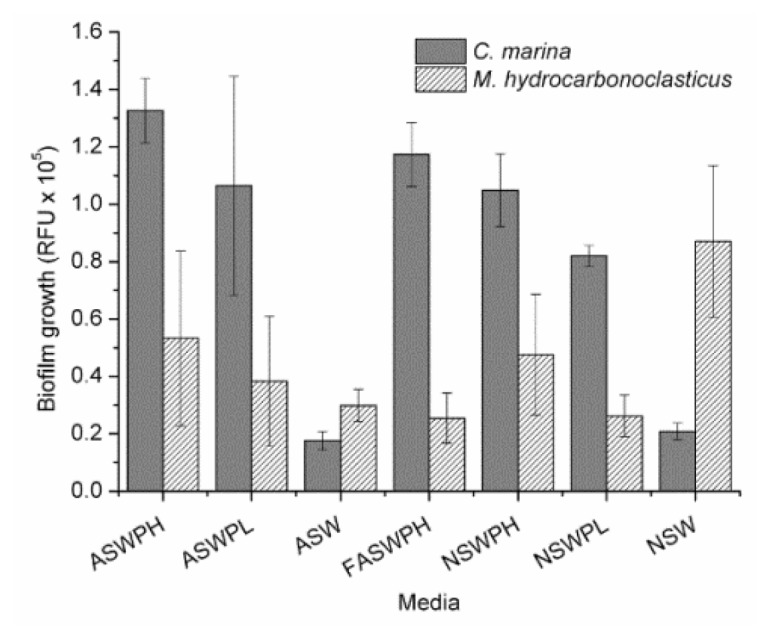
Biofilm formation after 24 h for both species, *M. hydrocarbonoclasticus* and *C. marina*, where: ASWPH, artificial sea water peptone high; ASWPL, artificial sea water peptone low; ASW, artificial sea water; FASWPH, filtered artificial sea water peptone high; NSWPH, natural sea water peptone high; NSWPL, natural sea water peptone low; and NSW, natural sea water.

The degree of *C. marina* biofilm formation within the various media was in good agreement with the planktonic growth data (Figure 2). This species formed similar biofilms at the same peptone concentrations for both ASW and NSW. Again, consistent with the cell growth assays, lower biofilm growth was observed for the treatments without any supplemented carbon source (*i.e.*, no peptone). In comparison to the growth rates (Table 1), there was generally good correlation between the test media/sterilisation and the biofilm formation, which demonstrated a good level of bioassay consistency for the ASW and NSW measurements.

In contrast, *M. hydrocarbonoclasticus* showed an interesting difference in behaviour when comparing planktonic cells to biofilm growth (Figure 2). Although the lowest planktonic growth was found to be in unsupplemented ASW and NSW (Table 1), biofilm formation was highest in NSW. The NSW is likely to contain organic nutrients, which are naturally occurring in coastal waters, thus providing a source of nutrition that could possibly supply sufficient energy for the initial biofilm formation. The ASW did not trigger the same response, possibly because no organic nutrients were present. Additionally, the *M. hydrocarbonoclasticus* American Type Culture Collection (ATCC) 49840 strain used in this study was isolated near a petroleum refinery from sediments collected from polluted areas [26], and it is worth noting that in the current study, NSW was collected from Southampton Water, which is one of the largest harbours in England. In addition, the Fawley refinery, which is the largest in the UK, is also located in the wider Southampton Water vicinity; therefore, water collected from this area may be favourable to this strain, resulting in enhanced biofilm formations. Therefore, it is possible that *M. hydrocarbonoclasticus* might be able to utilize organics that *C. marina* could not.

### 2.2. Protocol Verification

**Figure 3:**
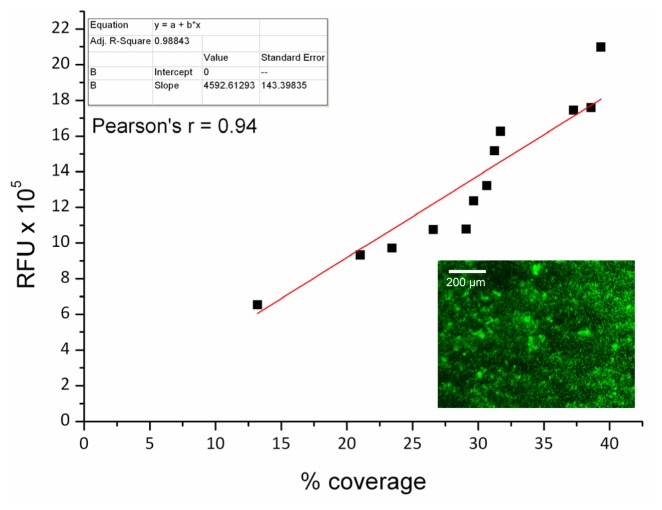
Syto^©^9 evaluation using two techniques, where *y*-axis data is obtained from the microplate-reader as a function of fluorescence intensity (RFU) and the *x*-axis is the same data obtained from epifluorescence microscopy, where ImageJ analysis was utilised to calculate the percentage of biofilm coverage on the 96-well plates. The bottom right insert illustrates a representative epifluorescence microscopy image of *C. marina* stained with Syto^©^9 and attached on the bottom of the well (1 h experiment).

For biofilm work, microscopy is largely used to confirm both qualitative and quantitative data regarding biofilm structure and amount. While microplate reader technology is becoming increasingly popular due to its rapid and high-throughput properties, microscopy is still necessary to validate fluorescence and imaging data obtained from the microplate reader. For this reason, a validation test was conducted in order to determine the correlation between the two techniques, using microscopy to corroborate the less established plate reader technique. A strong positive linear correlation was obtained between the fluorescence intensity measured using the microplate reader and bacterial surface coverage as determined by epifluorescence microscopy for *C. marina* (Figure 3).

The use of microplate readers to quantify biofilm biomass in itself is not novel. O’Toole *et al.* [27] developed an extremely useful rapid screen for biofilm based on multiwell crystal violet (CV) staining. CV is a basic dye, which binds to negatively charged surface molecules and polysaccharides in extra polymeric substances (EPS) [28,29]. Nevertheless, fluorescence staining has advantages over conventional biological stains: (i) fluorescence is more sensitive than adsorption, so that an elution step is not required; reducing the number of rinse steps, lessening the possibility of undesired biofilm removal; (ii) fluorescence can be measured directly from opaque surfaces (which are the relevant surfaces for marine and industrial application), unlike absorption, which requires a transparent surface or stain elution; (iii) there are numerous fluorescent stains available that are highly specific for individual biofilm constituents, such as DNA, carbohydrates, proteins and viability, thus by choosing the appropriate combination of fluorophores and filter sets, various components of interest can be quantified separately, but in a single step; and (iv) fluorescence staining is compatible with the microplate reader, epifluorescence and confocal microscopy.

### 2.3. *Chondrus crispus* Extracts and Bacterial Attachment

**Figure 4:**
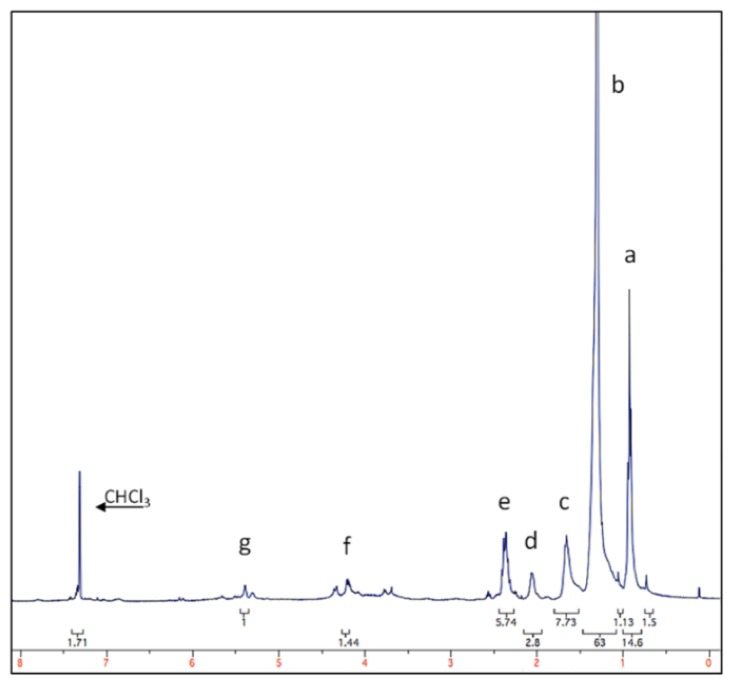
^1^H NMR spectrum of the *Chondrus crispus* organic solvent (toluene) extract.

*C. crispus* is commercially cultivated and available as a dried source and has been previously identified as having antifouling properties [19,23,30]. The ^1^H NMR spectrum of the toluene-soluble fraction of a crude ethanolic extract of dried *C. crispus* (designated CCT) was recorded. The analysed sample consists of a mixture of compounds to which it is not possible to assign any specific chemical structure. However the spectrum indicates a high content of aliphatic carbon chains with features typical of fatty acid esters (triglycerides) and a small amount of unsaturation (see Figure 4). No signals were observed at chemical shifts (δ) above 8 ppm, indicating a lack of free carboxylic acid groups (δ > 10 ppm). Fresh *C. crispus* has been shown to contain high levels of polyunsaturated fatty acid chains [31], so the apparent lack of unsaturation could result from the extended air drying process to which the seaweed has been subjected.

The major peaks in this spectrum can be assigned using the chemical shifts given in [32,33], (referenced to CHCl3 at δ = 7.28 ppm), Table 2.

Triglycerides are not known to exhibit marked AF effects; therefore, any anti-biofilm activity observed could be due to fatty acids produced by their hydrolysis in seawater or to other constituents of the crude extract not identifiable from this spectrum.

**Figure 5:**
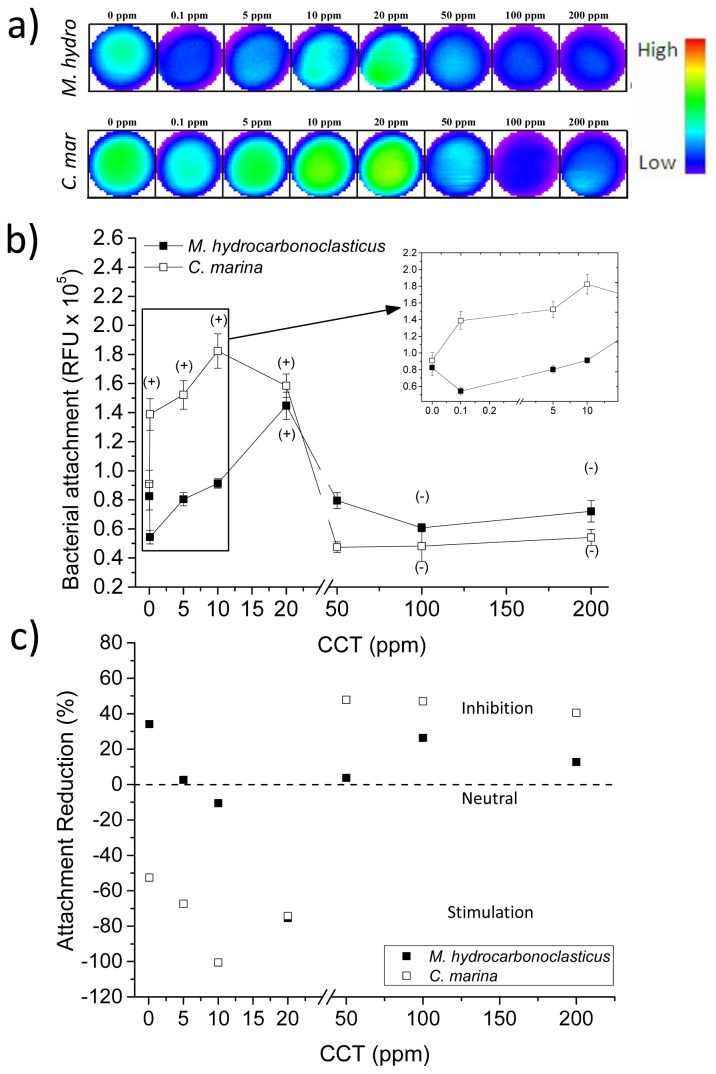
The effect of *C. crispus* on *C. marina* and *M. hydrocarbonoclasticus* biofilm accumulation, where: (**a**) is the well scan images of individual wells (scan matrix dimension: 30 × 30, equivalent to a resolution of 213 μm × 213 μm; scan width: 6 mm); (**b**) End point fluorescence intensity (units: relative fluorescent units, RFU). Positive/negative (+/−) symbols indicate a significant increase (+) or decrease (−) in the attachment of the species when compared with the control at 95% confidence intervals. Syto9: excitation λ = 485 nm, emission λ = 510 nm. Error bars ±SD; (**c**) Attachment reduction according to RFU values, as a percentage of the control (0%, dashed line), where negative values indicate increased attachment and positive values, reduction. CCT, *C. crispus* toluene soluble.

Figure 5a shows fluorescence intensity deriving from the attached cells in the individual wells (only one replica shown here) and their spatial distribution with respect to NP concentrations. Overall, for the CCT extract, bacterial attachment of *M. hydrocarbonoclasticus* was significantly increased when exposed to a concentration of 20 ppm (*p* < 0.001). However, the bacterial attachment was significantly reduced when *M. hydrocarbonoclasticus* was exposed to the higher CCT concentrations of 100 ppm and 200 ppm (*p* < 0.001 and *p* < 0.016, respectively; Figure 5b). For *C. marina*, attachment was significantly higher at low CCT concentrations, *i.e.*, 0.1 ppm, 5 ppm, 10 ppm and 20 ppm (*p* < 0.010, *p* < 0.002, *p* < 0.001 and *p* < 0.001, respectively; Figure 5b). However, at the higher concentrations of 50 ppm, 100 ppm and 200 ppm, attachment was significantly inhibited (*p* < 0.010, *p* < 0.020 and *p* < 0.031, respectively; Figure 5b). Although the overall accumulation for *C. marina* was reduced at high CCT concentrations, a concentration response was not observed, suggesting that this species was only affected by CCT concentrations between 20 ppm to 50 ppm and above (Figure 5b). The overall maximum attachment reductions were 35% and 50% for *M. hydrocarbonoclasticus* and *C. marina*, respectively (Figure 5c).

The reverse concentration response of enhanced *M. hydrocarbonoclasticus* and *C. marina*’s attachment at low CCT concentrations (Figure 5) may be explained by the presence of hydrocarbons, such as triglycerides, as shown in Figure 4, which are known to be used by both species as the sole carbon source [26,34]—found within seaweeds. In nature, nutrient concentrations in the biofilm may become limiting, due to consumption and diffusion within the biofilm [35,36]. To overcome this limitation, bacteria can consume algal exudates, while the algae may benefit from the recycling of nutrients produced by the bacteria, including carbon [37]. This beneficial mutual relationship between bacteria and the algae is reported to result in a high bacterial activity within the biofilm [38–40]. Cox *et al.* [41] have also recently reported that a methanolic *C. crispus* crude extract enhanced both Gram-negative and Gram-positive bacterial growth of *Listeria monocytogenes*, *Salmonella abony*, *Enterococcus faecalis* and *Pseudomonas aeruginosa*.

In an AF context, the literature describes specific AF activities of *C. crispus* extracts [18,19,23] and its by-products, such as hexose oxidase, which is used in a hydrogen peroxide-producing AF system [42]. In addition, Chambers *et al.* (2011) [23] reported that a crude ethanolic extract of dried *C. crispus* showed a greater AF effect than the extract derived from freshly harvested seaweed. However, the active components have yet to be identified, as the various NP extracts were not fully fractionated and assessed. Tannins are found in *C. crispus* extracts [41], and these are known to act against fouling organisms, such as barnacles [43]. An alternative antimicrobial mechanism has previously been proposed by Sullivan and Ikawa [44], who isolated and purified the enzyme, hexose oxidase, from *C. crispus* ([44–46]. Hexose oxidase is a copper-containing glycoprotein that catalyses the chemical reaction (Equation (1)):

(1) $$\text{D}-\text{glucose}+{\text{O}}_{2}↔\text{D}-\text{glucono}-1,5-\text{lactone}+{\text{H}}_{2}{\text{O}}_{2}$$

Metal component analysis of the enzyme has shown a copper content of 0.11%, *i.e.*, equivalent to 1100 ppm. Copper is known to be toxic to a variety of fouling organisms [47]; therefore, it may be hypothesised that the presence of hexose oxidase in *C. crispus* could negatively affect *M. hydrocarbonoclasticus* and *C. marina*, because of its copper content. Furthermore, enzymatic generation of hydrogen peroxide (H_2_O_2_) has been shown to have a promising AF efficacy [42,48]. However, in the current study, the use of toluene as a solvent would possibly limit the enzyme extraction.

### 2.4. Usnic Acid and Bacterial Attachment

**Figure 6:**
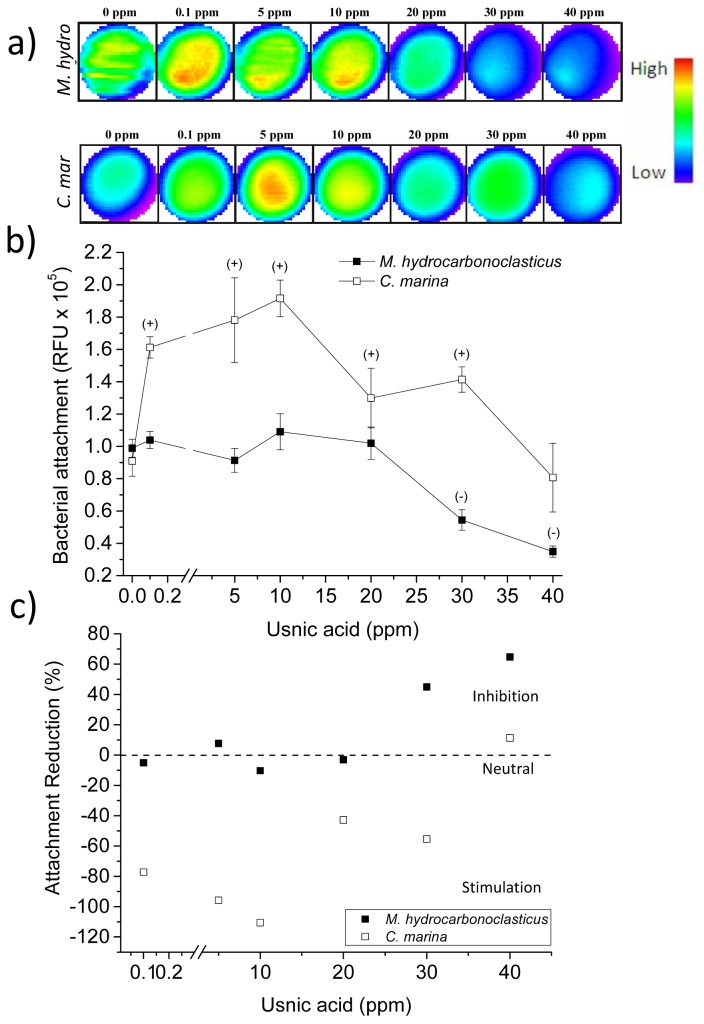
The effect of usnic acid on *C. marina* and *M. hydrocarbonoclasticus* attachment where: (**a**) is the well scan images of individual wells (scan matrix dimension: 30 × 30, equivalent to a resolution of 213 μm × 213 μm, scan width 6 mm); (**b**) End point fluorescence intensity (units: relative fluorescent units, RFU). Positive/negative (+/−) symbols indicate a significant increase (+) or decrease (−) in the attachment of the species when compared with the control at 95% confidence intervals. Syto9: excitation λ = 485 nm, emission λ = 510 nm. Error bars ±STDEV; (**c**) Attachment reduction according to RFU values, as a percentage of the control (0%, dashed line), where negative values indicate increased attachment and positive values, reduction.

The well scans in Figure 6a show more prominent localised fluorescence intensities for *M. hydrocarbonoclasticus* in comparison to *C. marina*. Overall, the bacterial attachment bioassays demonstrated that *M. hydrocarbonoclasticus* was not affected by the addition of usnic acid at the low concentrations of 0.1 ppm, 5 ppm, 10 ppm and 20 ppm. However, bacterial attachment was significantly inhibited in a concentration response way by usnic acid at concentrations of 30 ppm and 40 ppm (*p* < 0.022 and *p* < 0.001, respectively; Figure 6b). The highest usnic acid concentration caused the highest attachment reduction (65%) of all NPs tested in the current study for *M. hydrocarbonoclasticus* (Figure 6c). The effect of usnic acid on marine bacteria has not been reported previously. According to the literature, this compound is mostly active against Gram-positive species with little evidence of antimicrobial efficacy against Gram-negative bacteria [49]. The current results provide the first evidence of the effect of usnic acid on bacterial attachment for the Gram-negative marine bacterium, *M. hydrocarbonoclasticus*. For *C. marina*, no inhibitory effect was observed (Figure 6), rather an enhancement at almost all concentrations for usnic acid with an 80% to 110% increase in attachment for 0.1 ppm to 10 ppm (Figure 6c). We speculate that trace amounts of usnic acid, which is a quinone, might provide antioxidant benefits; it is also possible that there might be a signal effect similar to that in *P. aeruginosa*, where low concentrations of tobramycin stimulated biofilm growth [50]. While these hypotheses are interesting and potentially worth pursuing, they are outside the scope of work of the present study.

Usnic acid possesses antimicrobial activity against a number of planktonic Gram-positive bacteria [51], as well as antiviral activity. Although the mechanism of action of usnic acid against bacteria is still unknown, experimental evidence has illustrated that its antiviral activity is due to its ability to inhibit RNA transcription [52]. It has been shown that (+)-usnic acid and its isomer (−)-usnic acid are active against clinic isolates of *E. coli* and *E. faecium* (minimum inhibitory concentration MIC: 16 ppm) and clinical isolates of methicillin- or mupiricin-resistant *Staphylococcus aureus* (MIC: 16 ppm [49]). In the same study, it was shown that both isomers exhibited profound activity against pathogenic anaerobic *Bacteroides* sp. *Clostridium* sp. and *Propionibacterium* sp. (MIC: <16 ppm, 4 ppm and 2 ppm, respectively). Furthermore, Francolini *et al.* [24] reported an MIC of 32 ppm for *S. aureus*, while the Gram-negative *Pseudomonas aeruginosa* showed an MIC at 256 ppm. In the same study, the effect of usnic acid (loaded on a polymer) on initial bacterial attachment using flow cells was assessed for *S. aureus* and *P. aeruginosa*, where it was found that usnic acid did not affect biofilm formation for *P. aeruginosa*. However, it significantly affected biofilm morphology (shape and thickness), indicating that the molecule may have interfered with signalling pathways.

### 2.5. Juglone and Bacterial Attachment

**Figure 7:**
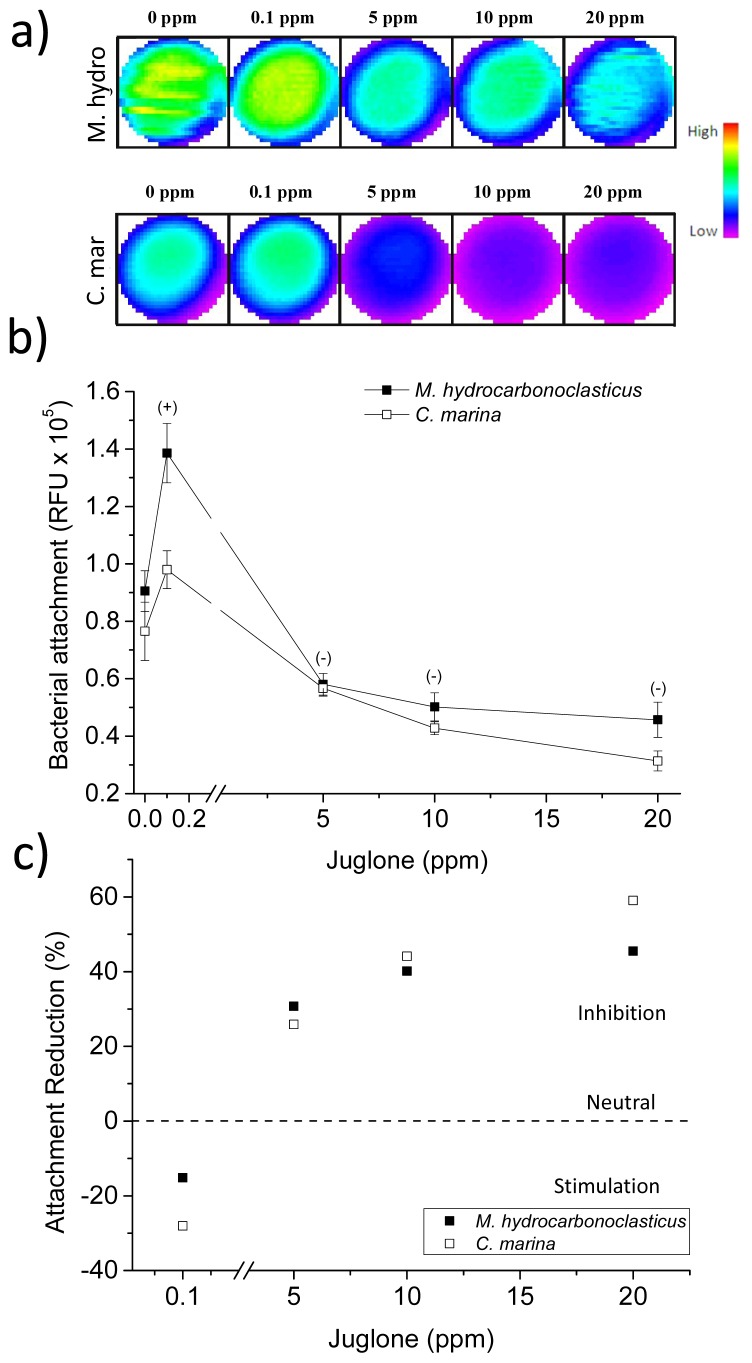
The effect of juglone on *C. marina* and *M. hydrocarbonoclasticus* attachment where: (**a**) is the well scan images of individual wells (scan matrix dimension: 30 × 30, equivalent to a resolution of 213 μm × 213 μm, scan width 6 mm); (**b**) End point fluorescence intensity (units: relative fluorescent units, RFU). Positive/negative (+/−) symbols indicate a significant increase (+) or decrease (−) in the attachment of the species when compared with the control at 95% confidence intervals. Syto9: excitation λ = 485 nm, emission λ = 510 nm. Error bars ±STDEV; (**c**) Attachment reduction according to RFU values, as a percentage of the control (0%, dashed line), where negative values indicate increased attachment and positive values, reduction.

In Figure 7, juglone was shown to give a clear dose-dependent concentration response effect inhibiting *M. hydrocarbonoclasticus* attachment at 5 ppm, 10 ppm and 20 ppm (*p* < 0.007, *p* < 0.005 and *p* < 0.004, respectively; Figure 7a,b). Similarly, *C. marina*’s attachment is significantly reduced by juglone in a concentration response manner at 10 ppm and 20 ppm (*p* < 0.050 and *p* < 0.035, respectively; Figure 7b). At the lowest juglone concentration (0.1 ppm), there is an apparent increase in attachment (although not statistically significant) for both species, similar to the *C. crispus* extract. Overall, at the highest concentrations, this NP inhibited attachment by about 45% and 55% for *M. hydrocarbonoclasticus* and *C. marina*, respectively (Figure 7c). The current study reports, for the first time, the use of juglone against marine biofilm forming bacteria and illustrates an activity against bacterial attachment. In the context of AF strategies against marine biofilms, juglone appears to be a promising compound, which will be further investigated.

Juglone is widely reported to be growth-stunting to many plants types, where it exerts an allelopathic effect by inhibiting specific enzymes needed for metabolic function [53]. Quinone-type compounds have been widely identified and used as anticancer, antibacterial or antimalarial drugs and, also, as fungicides and herbicides [54]. Additionally, herbal preparations derived from juglone extracts have been used as hair dyes and skin colorants, in addition to being applied topically for the treatment of acne, inflammatory diseases, ringworm and fungal, bacterial or viral infections. Their therapeutic effect has been attributed to quinone cytotoxicity [55]. The overall toxicity mechanism of naphthoquinones has still not been clearly established, especially for prokaryotes. However, in general, juglone is classified as a strong redox cycler with high potential to react with oxygen and its reactive species. Therefore, it interferes with vital cell processes, such as respiration, photosynthesis, cell division and membrane transport [56,57].

The antimicrobial activity of naphthoquinones has been reported in several studies [25,58–61]. Juglone has been shown to act against the marine bioluminescent species, *Vibrio fischeri*, and the dinoflagellate, *Glenodinium foliaceum* [25]. Using the Lumitox^®^ assay for *V. fischeri* (where the reduction of bioluminescence is linked to the inhibition of cellular activity, as a decreased rate of respiration), juglone exhibited bactericidal activity at concentrations as low as 0.005 ppm. In another study, the same naphthoquinone (5 hydroxy-1,4-napthoquinone) extracted from the heartwood of *Caesalpinia sappan* exhibited a good antibacterial performance against the intestinal Gram-positive bacterium, *Clostridium perfringens* [62]. Furthermore, a related US patent [63] claims solutions of juglone, or its derivatives in organic solvents as additives, for removing zebra mussels and quagga mussels from water intake pipes and various other underwater hard surfaces.

Overall, these results suggest that each of these terrestrial NPs can act as both an attachment promoter, as well as an inhibitor, depending on the NP concentration. This is supported by recently published studies, where it was reported that at high concentrations, antibiotics eradicate bacteria, while at low concentrations, biofilm formation is induced [50,64]; this phenomenon is often termed a hormetic response within the medical and toxicological context [65]. The results presented here, especially for juglone, appear to be promising, and we believe that, ultimately, a combination of surface texturing and chemistry will lead to the most effective AF performance.

### 2.6. Physico-Chemical Parameters

An understanding of an AF compound’s solubility in water is a necessity. Most frequently, this parameter is estimated by means of quantitative structure/activity relationships based on the log octanol-water partition coefficient (log*K*_ow_). It is an important parameter used in the assessment of environmental fate and transport of organic chemicals, because the octanol phase is a surrogate for the lipid phase or organic carbon content of the environmental compartments. For comparison, the physical chemistry parameters relating to solubility for the two terrestrial compounds and four organic antifouling biocide molecules (tolylfluanid, dichlofluanid, DCOIT (4,5-dichloro-*N*-octyl- 4-isothiazolin-3-one) (SeaNine™) and cybutryne) that are approved under the Biocidal Products Directive 98/8/EC can be seen in Table 3.

**Figure 8:**
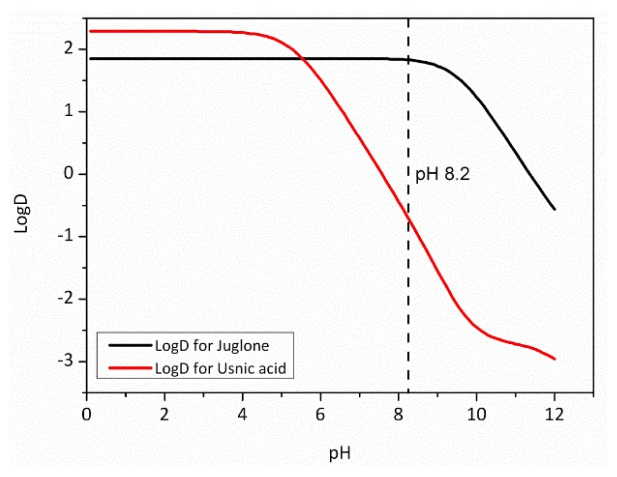
Log*D* values for juglone and usnic acid, where the vertical dashed line signifies the average sea water pH (8.2).

Log*P* (also referred to as log*K*_ow_) is the logarithm of the partition coefficient of the unionised compound between octanol and water phases at equilibrium. Log*D* is the logarithm of the distribution coefficient of an ionisable compound between octanol and water phases at defined pH values. The p*K*_a_ parameter is the negative logarithm of the acid dissociation constant. Calculated log*D* values for juglone and usnic acid over the pH range of 0–12 are plotted in Figure 8.

Partitioning of juglone into the water phase is low below pH 9, while the more easily ionized usnic acid increasingly enters the water phase at pH > 5. At seawater pH (average seawater pH: 8.2), usnic acid is predominately partitioned into the water phase, while juglone remains in the octanol phase. For polystyrene microtiter plates, adsorption of lipophilic organic substances on to the well walls will occur, depending on the log*P* and/or log*D* values. Generally, it is considered that chemicals with a log*P* of greater than three should be regarded as problematic within microtiter assays depending on the stability of the concentration over the experimental timescales [67,68]. Although both NP compounds have log*D* values at pH 8.2 lower than three (−0.65 and 1.83 for usnic acid and juglone, respectively), there is greater potential for juglone to have adsorbed on to the well surfaces to some extent, changing the concentration within the ASW.

The log*P* of 1.83, indicating a higher degree of hydrophilicity for juglone than for the commercial biocides, suggests that it has a lower potential for bioaccumulation and adsorption to sediments following release into the marine environment, as reported by [25]. In addition, the persistence of juglone has been shown to be dependent on the sterile status and light/dark conditions, for instance, a half-life of 12.7 h in sterile illuminated estuarine water *vs*. 87 h in non-sterile and dark conditions. By contrast, previously used commercial biocides, such as Diuron and Irgarol, have half-lives of 14 and 250 days, while their log*P* values are 2.8–3.95 and 2.8, respectively [69]. Log*P*, although it does not take pH into account, is often used as a relative indicator of the tendency of an organic compound to adsorb into marine sediments. Juglone, with a relatively low log*P* value, is known to have limited bioaccumulation and a short half-life [25,70], and thus, it could be considered an appropriate candidate compound for AF coatings. From an effective AF perspective, if usnic acid were to be incorporated into a coating and released, due to its low log*D*, the leachant would predominantly exist in the aqueous phase, leading to rapid dispersion and dilution in the marine environment. Juglone with a higher log*D* may exist at relatively greater local concentrations at the coating surface and, thus, resulting in a greater potency. It would therefore be a more effective NP candidate for AF coatings [70].

## 3. Materials and Methods

### 3.1. Natural Products

Dried commercially available *C. crispus* (carrageen, supplied by Carraig Fhada Seaweed, Ireland) was used for preparing a natural product extract according to [23]. The organic extract was further purified by dissolving in toluene and discarding any insoluble residue (e.g., salts), before recovering the extract by evaporation of the solvent. Nuclear magnetic resonance (NMR) analysis of the purified extract dissolved in chloroform-d (99.96 atom% D, Aldrich) was conducted using a Bruker DPX400 FT-NMR spectrometer.

**Scheme 1:**
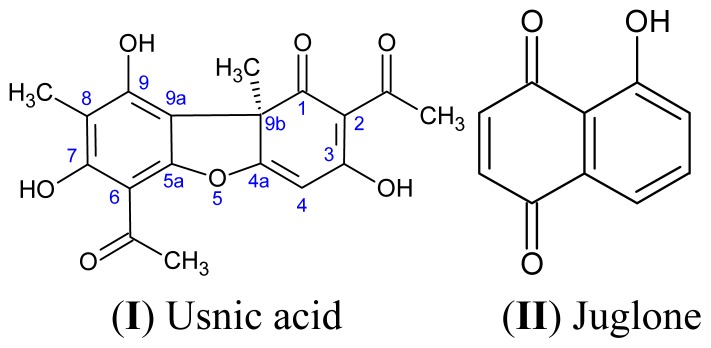
Molecular structures of the terrestrial natural products use in the current study.

A biologically active secondary metabolite identified in lichens of several species, including *Usnea* and *Cladonia*, is usnic acid [2,6-diacetyl-7,9-dihydroxy-8,9b-dimethyl-1,3(2*H*,9b*H*)-dibenzo-furandione]. Usnic acid (Scheme 1) is a yellow pigment that exists in two enantiomeric forms, (+)-usnic acid and (−)-usnic acid, depending on the projection of the angular methyl group at the chiral 9b position. While designated an acid, the molecule contains no carboxylic acid (–COOH) groups. However, the hydroxyl (–OH) groups all have some acidic character, with the –OH at position 3 being the most strongly acidic (p*K*_a_ 4.4), due to an inductive effect of the keto group [71]. The solubility of usnic acid in pure water is <0.01 g/100 mL [72], but will be higher in seawater (pH 8), owing to the ionisation of the –OH groups. Crude lichens have been used in folk medicine since ancient times to treat various medical conditions. One biologically active secondary metabolite identified in lichens of several species, including *Usnea* and *Cladonia*, is usnic acid [2,6-diacetyl-7,9-dihydroxy-8,9bdimethyl- 1,3(2*H*,9b*H*)-dibenzo-furandione].

Juglone [5-hydroxy-1,4-naphthalenedione] (Scheme 1) occurs naturally in all parts of the black walnut (*Juglans nigra*) tree, especially the nut husks and roots. Juglone is an inhibitor of respiration, which deprives other plants of the energy needed for metabolic activity, and plants grown near black walnut trees exhibit symptoms, such as foliar yellowing, wilting and eventual death [73]. Juglone is used in consumer products, such as herbal medicines and hair dye formulations, although it is potentially toxic to humans [74].

Both juglone and usnic acid were purchased from Sigma-Aldrich^®^ (AR grade, cat. Nos. H47003 and 329967, respectively). This work has only investigated the (+)-usnic acid enantiomer.

### 3.2. Marine Bacteria Test Species

The marine biofilm forming species used in this study were *Cobetia marina* (ATCC 25374) and *Marinobacter hydrocarbonoclasticus* (ATCC 49840), obtained from the American Type Culture Collection (ATCC). Both species have previously been used as models in studies on initial attachment of marine bacteria to surfaces [75–77]. Marine bacteria species are commonly cultured in marine broth, which includes peptone (P) as a carbon source recommended for ATCC species, in either sterilised natural sea water (NSW) or artificial sea water (ASW). Both species were kept as frozen stock aliquots in sea salts (ASW, Sea Salts S9883, Sigma Aldrich, St. Luis, MO, USA) plus 18 g L^−1^ peptone, abbreviated as SSP [77], with 10% glycerol added and preserved at −80 °C. The cryopreserved culture stocks were plated on marine agar (BD DifcoTM Marine Agar 2216, Franklin Lakes, NJ, USA) placed in a cooled incubator (Binder KB115, Binder, Mittleren, Germany) at 28 °C. Young colonies were formed within 42 h after incubation. A single colony was then picked using a sterile inoculation loop and transferred into sterile SSP, where the bacteria were left to grow overnight. The recommended temperature range for both species from ATCC is 26–30 °C.

### 3.3. Culture Conditions and Choice of Media

New cultures of *M. hydrocarbonoclasticus* and *C. marina* from frozen stock aliquots were plated on marine agar for 3 days. After 3 days, individual colonies were inoculated in SSP and incubated at 28 °C under agitation (80 rpm), overnight. The cultures were then washed in order to avoid any medium carry over using the following steps: 40 mL of the overnight cultures were placed into 50 mL centrifuge tubes, and the cells were centrifuged at 4000 rpm for 8 min at 6 °C. Once the cells were centrifuged down, creating a pellet at the bottom of the centrifuge tube, the supernatant (medium) was discarded, and sterile ASW was introduced. The cells were then re-suspended, using a vortex mixer, in ASW and re-centrifuged.

The differences between ASW and NSW compositions may vary greatly, due to naturally occurring nutrient seasonality observed in the NSW, plus variable salinity and pollutant levels at the sampling location. NSW for this study was collected from Southampton Water, summer, 2011. For laboratory-based assays, ASW is generally considered to provide greater controllability over the aforementioned water properties, and it was therefore decided to conduct a series of tests comparing the different experimental media, *i.e.*, NSW *vs*. ASW, and the levels of carbon typically used in bacterial bioassay testing. The sterilisation procedure was also assessed in the current experiments, *i.e.*, media autoclaved *vs*. sterile filtered through a 0.2 μm pore size (Steritop™ Millipore^®^ Filter Units, Billerica, MA, USA). This was done for two main reasons: (1) autoclaving ASW (without peptone) produced precipitates in the solution and (2) preliminary experiments illustrated a significant change in the medium’s pH (the solution becoming more alkaline) after autoclaving. NSW was collected from Southampton Water, UK, in May, 2010, and glass micro fibre filters GF/F were used to remove coarse particulates prior to the sterilisation procedures. The complete experimental matrix of the media tested can be seen in Table 4.

#### 3.3.1. Planktonic Growth on Different Media

First, we assessed growth on different media in order to choose the most appropriate for subsequent routine testing. Wells of a 96-well plate (optical bottom plated with polymer base, black, Nunc™) were conditioned by the addition of 190 μL of the experimental media 1 h before bacterial inoculation. Following the well conditioning, 10 μL of washed cells were inoculated into the wells to achieve a final volume of 200 μL per well. Seven different experimental media were tested, as outlined in Table 4.

Five replicas for each treatment were performed. Four replicas of un-inoculated wells were also used as blanks for possible procedural contamination and background signals. Cell growth experiments were performed for a total duration of 24 h at 28 °C, and absorbance at λ = 595 nm (commonly referred to as optical density, OD_595_) was measured using a microplate reader (FLUOstar Omega, BMG LABTECH, Offenburg, Germany). The optical density (OD_595_) was measured every 15 min. The software used was Mars Data Analysis Software, Version 2.00 (Omega, BMG LABTECH, Offenburg, Germany).

#### 3.3.2. Biofilm Growth under Different Medium Conditions Using Nucleic Acid Staining

To quantify the amount of biofilm at the end point of the growth period, the media in the 96-well microplates were removed, and each well was individually rinsed (by pipetting) twice with ASW in order to remove planktonic cells. After the washing steps, the biofilm was stained with Syto^©^9 (Molecular Probes^®^, Eugene, OR, USA), according to the manufacturer’s instructions, *i.e.*, 2 μL of the Syto^©^9 (provided at 5 mM in 100% DMSO) per 1 mL of Phosphate Buffer Saline (pH 7.4). Syto^©^9 is a green fluorescent intercalating membrane permeable stain, which is expected to stain all cells. Following the staining procedure, the plates were incubated in the dark at 28 °C for 20 min. The microplate reader multichromatic filters were calibrated to suit the excitation and emission wavelengths of Syto^©^9. Syto^©^9 fluorescence intensity and imaging were used to quantify biofilm biomass (see the below details on wavelengths).

Following the incubation period, the wells were again washed twice with ASW to remove excess stain. Finally, 100 μL of ASW were added to the wells to prevent biofilm dehydration during the measurements. The microplate reader was utilised for direct end point measurements (relative fluorescence units, RFU) to allow high-resolution analysis of fluorescence intensity, which was conducted using λ_EX_ = 485 nm (for excitation) and λ_EM_ = 510 nm (for emission).

### 3.4. Attachment Bioassays

The 96-well microplates (optical bottom plated with polymer base, black, Nunc™) were inoculated with 200 μL of ASW for 1 h prior to bacterial inoculation in order to condition the wells. After 1 h, the wells were emptied, and 199 μL of the prepared bacteria (washed according to the previous section and re-suspended in ASW at a starting concentration of OD_595_ = 0.2) were inoculated in each well. In order to fully dissolve the natural products (NPs), 100% dimethyl sulfoxide (DMSO) was used as the solvent to produce a stock solution of 40,000 ppm, while serial dilutions in DMSO (100 vol%, all percentages by volume) followed to give the final concentrations listed in Table 5. The concentrations used were comparable with those reported in the antifouling literature, typically in the order of 1 ppm to 1000 ppm [78,79]. A 1 μL aliquot from each NP stock solution was added to the wells, giving a final well volume of 200 μL. Using the NP dissolved in a small volume (1 μL) of DMSO was found to be the preferred option, as it allowed a low final DMSO concentration (0.5%) in the wells. DMSO can be bacteriostatic at high concentrations of 12.5%–20% [80,81]; therefore the lower its concentration, the lower the risk of bacterial toxicity due to this solvent, rather than the NP itself. Control wells with no NP validated that 0.5% DMSO did not affect bacterial attachment. Each NP concentration was replicated five times, while for each concentration, three uninoculated wells served as blanks. Controls included: bacteria in ASW and bacteria in ASW plus 0.5% DMSO. In Table 5, the complete experimental matrix for the NPs is shown.

Following bacteria inoculation and NP additions, the 96-well plates were incubated for 1 h at 28 °C under static conditions. The attachment assay was carried out for 1 h [77]. After the incubation time, OD_595_ measurements were taken to establish the planktonic growth, and no significant differences were found between the treatments (*p* > 0.05). Then, the NPs and controls in the 96-well plates were removed, and each well was individually rinsed (by pipetting) twice with ASW in order to remove any planktonic cells. Staining with Syto^©^9 was followed (as explained in the previous section) to achieve bacterial attachment quantification. In addition, the well scanning capability of the microplate reader was used for spatial visualization of fluorescence intensity emitted by newly attached bacteria in individual wells, allowing for identification of heterogeneous biofilm structures and their distribution (e.g., whether there is marked attachment on the well walls, which is often considered an issue in well plate assays). Each well was scanned to produce an image of the newly attached formations (scan matrix dimension: 30 × 30, equivalent to a resolution of 213 μm × 213 μm, and a scan width of 6 mm). The main advantage of the imaging utility is its sufficient sensitivity to identify biofilm features, as well as the plethora of sampling points within the well, providing a very accurate measurement representative of the entire well. Importantly, the fluorescence signals from the walls can be excluded, providing, in this way, accurate measurements exclusively from the well’s bottom. The percentage of attachment reduction (*R*%) was determined by the following expression (Equation (2)):

(2) $$R\%=\left(\frac{\left(Con-Exp\right)}{Con}\right)\times 100$$

where, *Con* = control (*i.e.*, no compound added) and *Exp* = experimental (*i.e.*, with compounds).

### 3.5. Syto^©^9 Validation (Microplate Reader-Epifluorescence Microscopy)

Separate attachment experiments were conducted (according to the above protocol) to compare/validate microplate reader measurements of Syto^©^9 against epifluorescence microscopy. At the experimental end point (1 h) and following the Syto^©^9 staining, the 96-well plates were placed under an epifluorescence microscope for visualisation using the 10× magnification objective lens (EVOS*fl*, AMG, Life Technologies, Carlsbad, CA, USA) and the standard green-fluorecent-pigment (GFP) light-emitting diode (LED) light cube (λ_EX_: 370 nm–λ_EM_: 525 nm). The images were acquired using the microscope’s built-in camera (a high sensitivity interline charge-coupled device camera). Five replica images were taken for each well and within a defined region of interest (ROI), avoiding the well walls, resulting in a total of *N* = 45 per treatment. Image analysis was conducted using ImageJ (Biophotomics, Available online: http://rsbweb.nih.gov/ij/plugins/mbf/index.html), where images were transformed into a binary format, *i.e*., either 0 or 255, and the percentage of coverage of attached bacteria was quantified. The calculated percentage coverage from each well was then plotted against its equivalent datum obtained from the plate reader, *i.e.*, average fluorescence intensity (RFU).

Once the percentage of coverage of bacteria attached on the surface (bottom of the well) was calculated for each well, data were plotted against the average fluorescence intensity (RFU) obtained from the plate reader for the same well.

### 3.6. Physico-Chemical Parameters

The physico-chemical parameters, solubility, Log*P*, p*K*a^1–3^ and Log*D* at pH 8.2, for the four organic antifouling biocide molecules: tolylfluanid, dichlofluanid, DCOIT (SeaNine™, DOW Chemical Company, Midland, MI, USA) cybutryne and the two terrestrial NPs, juglone and usnic acid, were calculated using the online version of ACD/Labs Algorithm Version: v5.0.0.184 (https://ilab.acdlabs.com/iLab2/index.php).

### 3.7. Statistical Analysis

Differences in OD_595_ and RFU values between experimental and controls were assessed by applying one-way ANOVA. To establish the homogeneity of variances, Levene’s test of equal variances was applied. Where homogeneity of variances was not met, the non-parametric Kruskal-Wallis test was applied. Statistical analysis was performed using SPSS software (version 17.0, IBM, New York, NY, USA). Differences were considered statistically significant for *p* < 0.05.

## 4. Conclusions

The toluene soluble part of *C. crispus* (CCT) was found to inhibit bacterial attachment at concentrations of 50–200 ppm for *C. marina* and 100–200 ppm for *M. hydrocarbonoclasticus*. However, low CCT concentrations appeared to have promoted *M. hydrocarbonoclasticus*’ attachment, suggesting an alternative activity of this extract in a concentration-dependent manner. Usnic acid was found to be active against *M. hydrocarbonoclasticus*, deterring biofilm attachment at concentrations of 30 and 40 ppm, while *C. marina* was not inhibited by this natural product. The best anti-biofilm performance was observed for the terrestrial pure compound, juglone. This NP inhibited both *M. hydrocarbonoclasticus*’s and *C. marina*’s attachment, in a concentration response manner, at concentrations as low as 5 ppm to 20 ppm. In addition to existing AF bioassays against marine biofilms (such as OD, visual observations or simple staining), this study has explored the use of nucleic acid staining for the *in situ* quantification of newly attached bacteria (1 h) and successfully correlated the two principal techniques for bacterial and biofilm work, the microplate reader and epifluorescence microscopy.

## Figures and Tables

**Table 1:** Growth rates and doubling times for both species, *Marinobacter hydrocarbonoclasticus* (*M. hydro*) and *Cobetia marina* (*C. mar*), during the exponential growth phase using absorbance λ= 595 nm (Optical Density at λ = 595 nm (OD_595_)), where: ASWPH, artificial sea water peptone high; ASWPL, artificial sea water peptone low; ASW, artificial sea water; FASWPH, filtered artificial sea water peptone high; NSWPH, natural sea water peptone high; NSWPL, natural sea water peptone low; and NSW, natural sea water.

Species		ASWPH	ASWPL	ASW	FASWPH	NSWPH	NSWPL	NSW
*M. hydro*	Growth rate (h^−1^)	0.115 ± 0.008	0.091 ± 0.006	0.022 ± 0.010	0.113 ± 0.007	0.109 ± 0.009	0.090 ± 0.002	0.013 ± 0.002
	Doubling time (h)	6.03 ± 0.39	7.59 ± 0.52	35.66 ± 14.80	6.11 ± 0.36	6.35 ± 0.53	7.63 ± 0.22	53.38 ± 8.41

*C. mar*	Growth rate (h^−1^)	0.079 ± 0.004	0.072 ± 0.002	0.055 ± 0.006	0.075 ± 0.002	0.073 ± 0.002	0.075 ± 0.002	0.06 ± 0.010
	Doubling time (h)	8.77 ± 0.47	9.54 ± 0.30	13.77 ± 2.25	9.21 ± 0.31	9.39 ± 0.30	9.15 ± 0.27	11.29 ± 2.51

**Table 2:** Assigments of ^1^H NMR chemical shifts according to Guillén and Ruiz [32,33].

Peak	ppm	Group
a	0.896	–CH_3_
b	1.272	–(CH_2_)*_n_*–
c	1.625	–OCO–CH_2_–CH_2_–
d	2.027	–CH_2_–CH=CH–
e	2.331	–OCO–CH_2_–
f	4.168	–CH_2_–OCOR
g	5.360	–CH=CH–

**Table 3:** Calculated physico-chemical parameters (using Advanced Chemistry Labs Algorithm Version: v5.0.0.184 (Available online: https://ilab.acdlabs.com/iLab2/index.php, accessed on 20 June 2013) for natural products (NPs) used in the current work compared with approved commercial biocides.

Parameters	Usnic acid	Juglone	Tolylfluanid	Dichlofluanid	DCOIT (SeaNine™)	Cybutryne
Solubility 1 (ppm)	420 *	160 *	39	58	63	390
Log*P*^2^	2.29 (RI = 0.63)	1.85 (RI = 0.88)	3.58 (RI = 0.82)	3.32 (RI = 0.80)	4.59 (RI = 0.62)	3.45 (RI = 0.75)
p*K* a1 3	5.3 ± 0.8	9.5 ± 0.4	−5.06 ± 0.5	−5.37 ± 0.5	−6.09 ± 0.6	4.12 ± 0.1
p*K* a2 3	9.0 ± 0.9		−16.79 ± 0.7	−16.89 ± 0.7		−2.55 ± 0.2
p*K* a3 3	12.0 ± 0.9					
Log*D* at pH 8.2	−0.65	1.83	3.58	3.32	4.59	3.45

1 In pure H_2_O;

2 Pharma Algorithms (AB)/Log*P* v2.0;

3 ACD Labs algorithm.

* The calculated solubility for both NPs (in deionised water) appears to be much higher than observed in the current work and in the literature (e.g., [66]). The solubility of usnic acid in water is highly dependent on pH, which may not have been controlled in practical studies.

RI = Reliability Index.

**Table 4:** Experimental media for *C. marina* and *M. hydrocarbonoclasticus*. ASW, artificial sea water; NSW, natural sea water; P, peptone; H, high concentration (18 g L^−1^); and L, low concentration (9 g L^−1^). SSP, sea salts and peptone.

Water	Peptone (g L^−1^)	Sterilisation	Acronym
ASW	18	Autoclaved	ASWPH *
	9	Autoclaved	ASWPL
	0	Autoclaved	ASW
	18	Filtered (0.2 μm)	ASWPH

NSW	18	Filtered (0.2 μm)	NSWPH
	9	Filtered (0.2 μm)	NSWPL

	0	Filtered (0.2 μm)	NSW

* ASWPH = SSP.

**Table 5:** Natural products (NP) used to assess bacterial attachment where, JUG, juglone; UA, usnic acid; CCT, *C. crispus* toluene soluble. Units: ppm = μg mL^−1^.

NP	Concentration (ppm)
CCT	0.1, 5, 10, 20, 50, 100, 200
UA	0.1, 5, 10, 20, 30, 40
JUG	0.1, 5, 10, 20
